# Unplanned Reoperation within 30 Days of Fusion Surgery for Spinal Deformity

**DOI:** 10.1371/journal.pone.0087172

**Published:** 2014-03-04

**Authors:** Zheng Li, Jianxiong Shen, Guixing Qiu, Haiquan Yu, Yipeng Wang, Jianguo Zhang, Hong Zhao, Yu Zhao, Shugang Li, Xisheng Weng, Jinqian Liang, Lijuan Zhao

**Affiliations:** Department of Orthopaedic Surgery, Peking Union Medical College Hospital, Chinese Academy of Medical Sciences & Peking Union Medical College, Beijing, China; University of Louisville, United States of America

## Abstract

No recent studies have analyzed the rates of or reasons for unanticipated revision surgery within 30 days of primary surgery in spinal deformity patients. Our aim was to examine the incidence, characteristics, reasons, and risk factors for unplanned revision surgery in spinal deformity patients treated at one institution. All patients with a diagnosis of spinal deformity presenting for primary instrumented spinal fusion at a single institution from 1998 to 2012 were reviewed. All unplanned reoperations performed within 30 days after primary surgery were analyzed in terms of demographics, surgical data, and complications. Statistical analyses were performed to obtain correlations and risk factors for anticipated revision. Of 2758 patients [aged 16.07 years (range, 2–71), 69.8% female] who underwent spinal fusion surgery, 59 (2.1%) required reoperation within 30 days after primary surgery. The length of follow up for each patient was more than 30 days. Of those that required reoperation, 87.0% had posterior surgery only, 5.7% had anterior surgery, and 7.3% underwent an anteroposterior approach. The reasons for reoperation included implant failure (n = 20), wound infection (n = 12), neurologic deficit (n = 9), pulmonary complications (n = 17), and coronal plane imbalance (n = 1). The risk factors for reoperation were age, diagnosis, and surgical procedure with osteotomy.

## Introduction

Patients with spinal deformity usually present with symptoms such as visible deformity, pain, progression of deformity, sagittal or coronal imbalance, and/or neural compromise [1]. Fusion surgery for spinal deformity is intended to be the final therapeutic intervention in the management of this condition [2]. The goals of surgical treatment are to obtain a stable and solid fusion after a safe and optimal 3-dimensional correction of the spinal deformity [3]. Achieving these goals should also improve the patients' quality of life in the long term, compared to those who did not undergo surgical treatment [4]. Optimal management of spinal deformity continues to challenge both patients and surgeons. Despite recent improvements in the efficacy and safety of spinal fusion, complications following surgical correction of scoliosis deformity remain a reality [5], and various potential problems requiring further surgical intervention may develop in the immediate postoperative period or over time [6].

Unplanned reoperation within 30 days of primary surgery has recently been suggested as a useful quality marker in hospitals performing spinal surgery [7]. Reoperation is associated with poor clinical outcomes, including higher risk of complications and implant failures [6], [8]. In addition, the costs and the time associated with hospitalization for unplanned reoperation patients have been increasing [9]. A previous study reported that 7.5% of 452 cases of idiopathic scoliosis correction required reoperation [10], [11]. Another study documented a 3.9% overall reoperation rate [12]. In cases of adult spinal deformity, the cumulative reoperation rate has been found to be 25.8% [13]. The reasons for reoperation include infection, pseudarthrosis, adjacent segment problems, implant failure, neurologic complications, and curve progression [11]. To our knowledge, however, the rate and causes of unplanned reoperation within 30 days after primary surgery have not been reported.

The objective of this study was, therefore, to determine the incidence and factors contributing to unplanned reoperation within 30 days of fusion surgery for spinal deformity in our department.

## Results

Between 1998 and 2012, a total of 2758 consecutive patients underwent spinal fusion for spinal deformity at our institution. The mean age at the time of initial surgery was 16.07±8.3 years (range, 2–71 years). Females made up 69.8% of the cases (n = 1925), and males made up 30.2% of the cases (n = 833). The majority of patients (n = 2400, 87.0%) were treated using a posterior approach and instrumented spinal fusion. A total of 202 (7.3%) patients underwent a combined anterior and posterior spinal fusion, and 156 (5.7%) had an instrumented anterior spinal fusion. There was no overall difference between the revision and non-revision groups with respect to age and gender (Table 1).

**Table 1:** Patient Characteristics of Cohort.

	Reoperation (n = 59)	No operation (n = 2699)	p
Age (range)	17.86±9.0 (2–55)	16.07±8.2 (2–71)	p>0.05
Sex			p>0.05
Female	33	1870	
Male	26	829	
Max cobb	69°±16°	68°±1.9°	p>0.05
Mean levels fused	11.3±2.9	11.3±3.9	p>0.05

### Reasons for Reoperation

Of the 2758 patients identified as having primary surgery for spinal deformity, 59 patients (2.1%) underwent reoperation within 30 days of primary surgery for scoliosis. Table 2 illustrates the percentages of patients in the reoperation group and the cohort as a whole with respect to the reason for revision.

**Table 2:** Reoperation Reasons.

Reason for Reoperation	Reoperations (%)	Total patients (%)
implant failure	20/59 (33.9%)	20/2758 (0.73%)
infection	12/59 (20.3%)	12/2758 (0.44%)
neurologic deficit	9/59 (15.3%)	9/2758 (0.37%)
pulmonary complications	17/59 (28.9%)	17/2758 (0.62%)
coronal plane imbalance	1/59 (1.7%)	1/2758 (0.04%)
Total	59/59 (100%)	59/2758 (2.1%)

**Figure 1:**
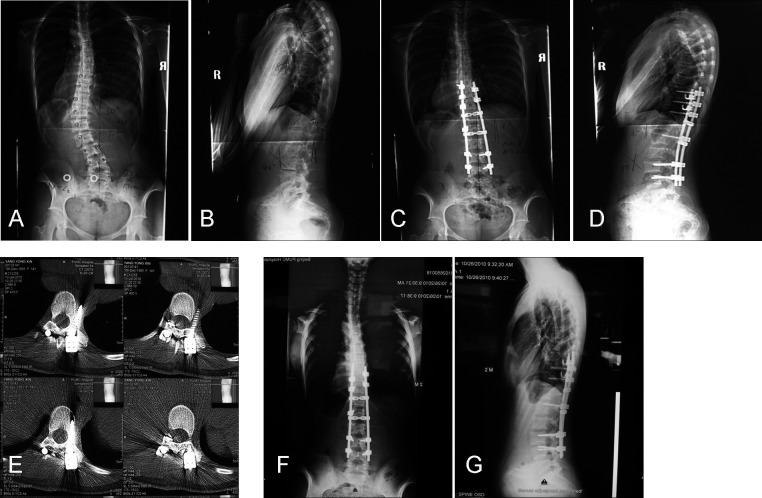
Patient is a 15 year-old female with Marfan syndrome with scoliosis. A and B, Standing preoperative anteroposterior and lateral radiographs. C and D, Standing anteroposterior and lateral radiographs 4 days after operation. E, Magnetic resonance images, showing improper implant location. F and G, Standing anteroposterior and lateral radiographs 4 days after reoperation.

**Figure 2:**
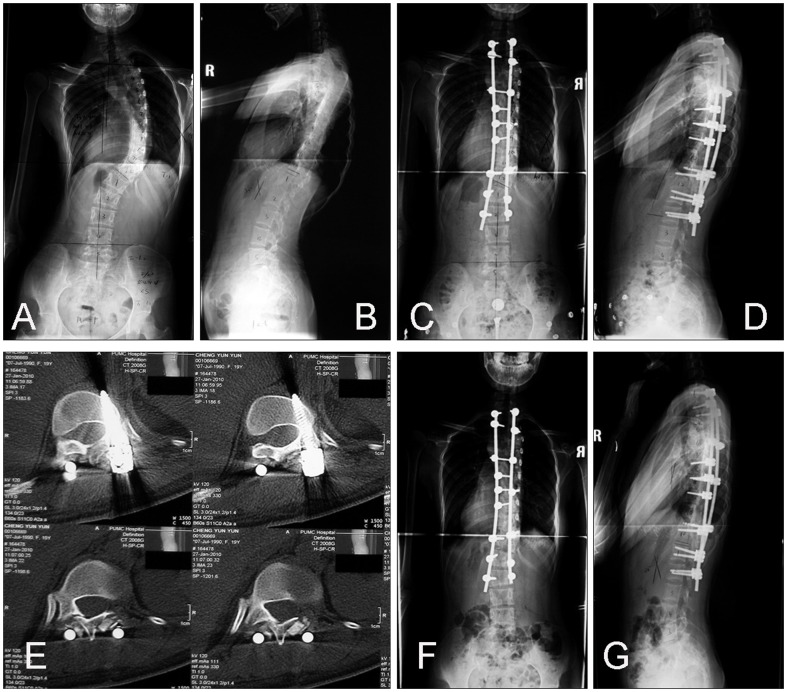
Patient is a 20 year-old male with congenital scoliosis. A and B, Standing preoperative anteroposterior and lateral radiographs. C and D, Standing anteroposterior and lateral radiographs 4 days after operation. E, Magnetic resonance images showing improper implant location. F and G, Standing anteroposterior and lateral radiographs 5 days after reoperation.

**Figure 3:**
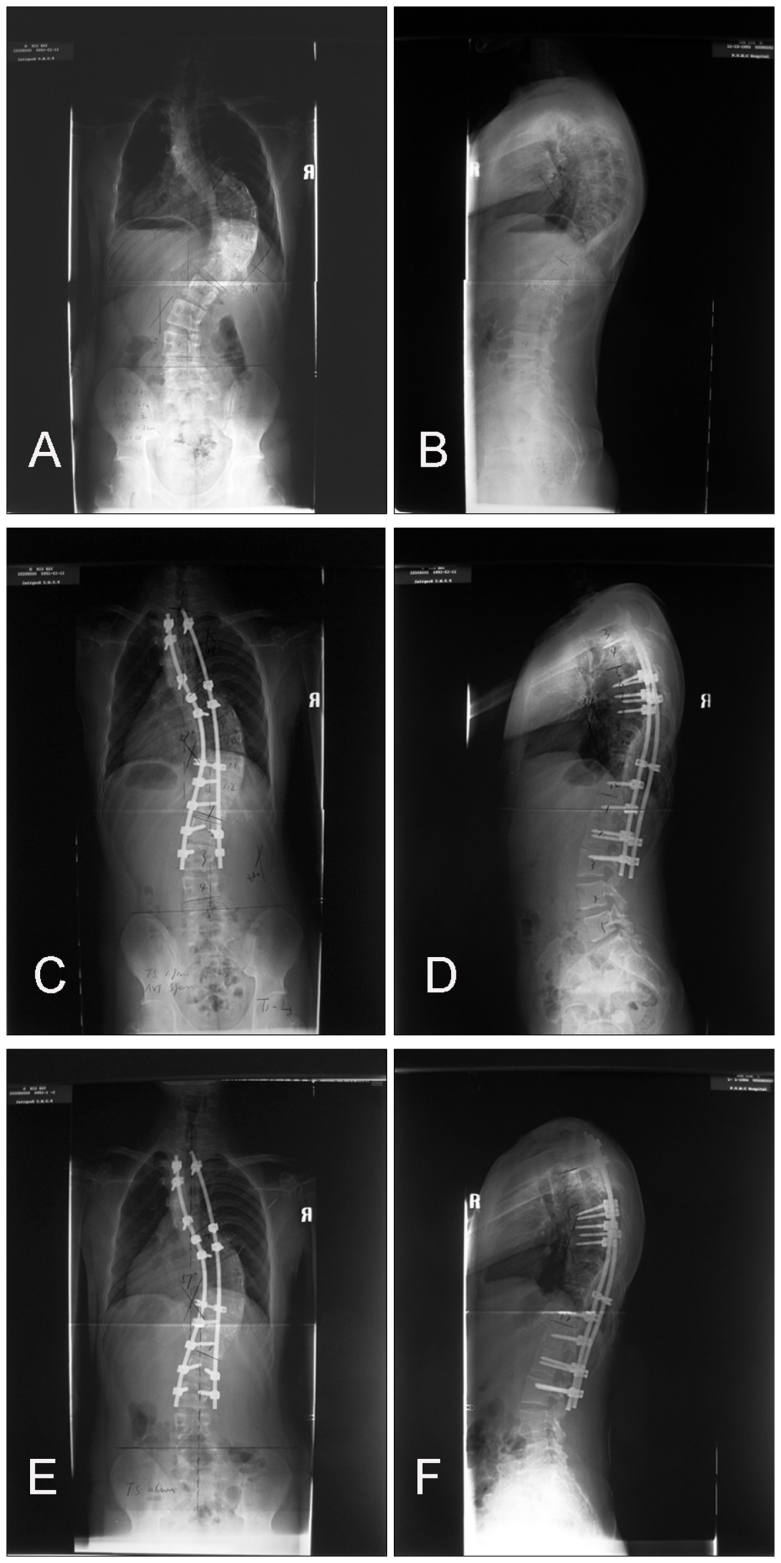
Patient is a 16 year-old female with neuromuscular scoliosis. A and B, Standing preoperative anteroposterior and lateral radiographs. C and D, Standing anteroposterior and lateral radiographs 4 days after operation showing pedicle screw loosening. E and F, Standing anteroposterior and lateral radiographs 5 days after reoperation.

Twenty repeat operations were performed due to implant-related failures. Of these, 6 (30%) were due to pullout of the hooks, 6 were because of improper implant location, 6 were due to loosening of pedicle screws, and 2 were due to screw cap loosening (Fig. 1, 2, and 3).

Infection was only noted in patients who underwent posterior spinal instrumentation and fusion as the index procedure; no infections occurred after anterior spinal instrumentation. This difference was significant (p<0.001). In all, 59 reoperations due to infections were performed in 12 patients. Eight of these 12 cases were due to deep wound infections, and four were due to superficial wound infections. The implants were not removed in any of these patients following the reoperation.

Nine revisions were performed because of the patient's neurologic deficit, 6 revisions were due to paraplegia, and 3 revisions were due to nerve root injury. Of the 6 patients who presented with paraplegia, 4 presented with complete paralysis of the lower extremity, and 2 presented with incomplete paralysis of the lower extremity.

Reoperations were performed in 17 patients due to pulmonary complications. Eight of these patients returned to the operating room for hydrothorax, 5 for hemothorax, 2 for pneumothorax and 2 for chylothorax.

One revision was performed for coronal plane imbalance.

### Sex, Age, Diagnosis and Surgical Approach

**Figure 4:**
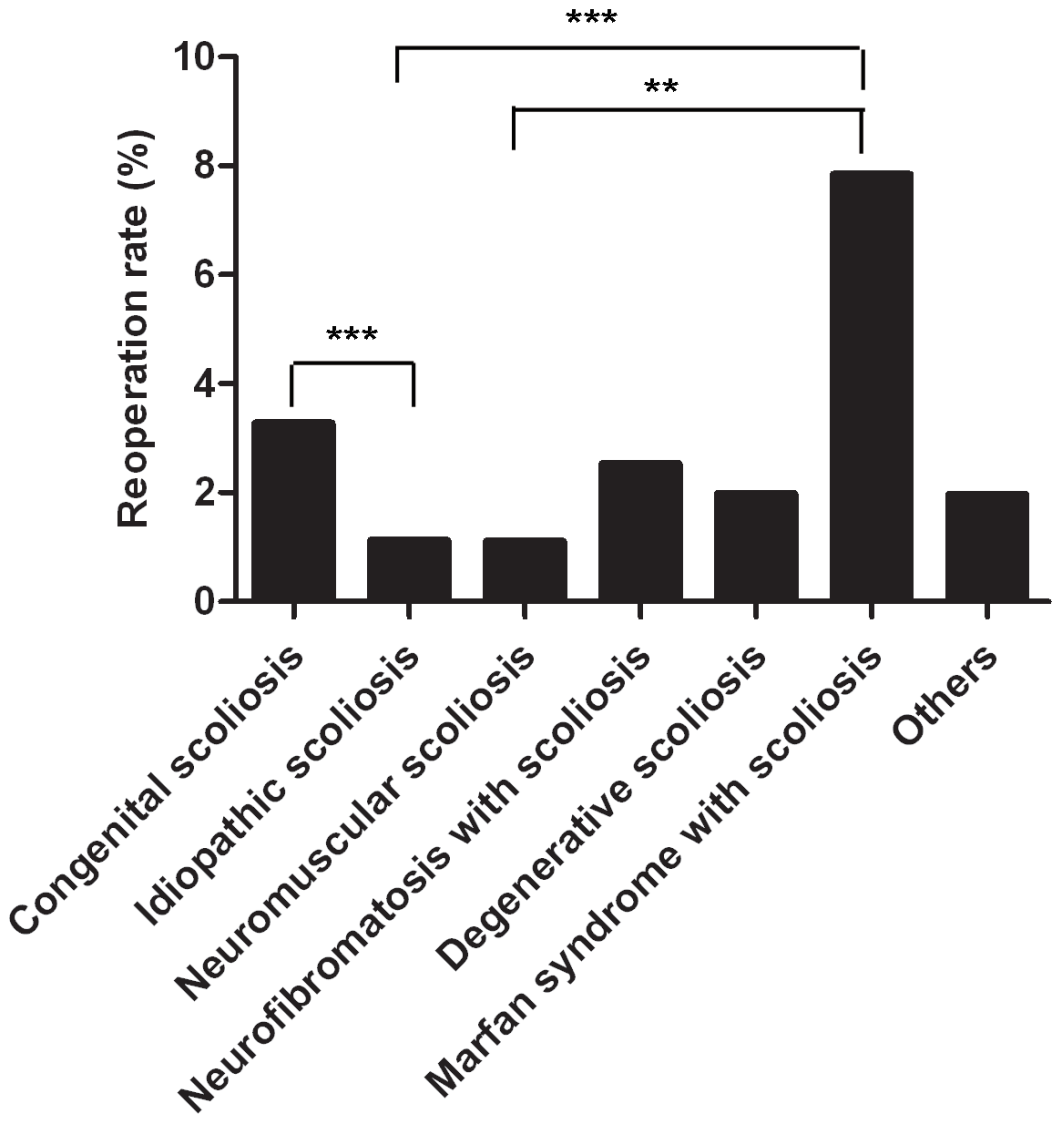
Diagnosis and Reoperation Rates. The statistically significant differences compared with the control are noted as *p<0.05, **p<0.01 and ***p<0.001. Others include syndrome-related scoliosis, ankylosing spondylitis, achondroplasia with scoliosis.

The breakdown of the primary diagnoses is as follows: 1255 (45.5%) IS, 1039 (37.8%) congenital scoliosis, 182 (6.6%) neuromuscular scoliosis, 79 (2.9%) neurofibromatosis scoliosis, 101 (3.7%) degenerative scoliosis, 51 (1.8%) Marfan syndrome with scoliosis and 51 (1.8%) other (syndrome-related scoliosis, ankylosing spondylitis, achondroplasia with scoliosis). Patients with Marfan syndrome with scoliosis had a much higher rate of reoperation (7.84%) and had a significantly higher rate of reoperation when compared with the idiopathic scoliosis group (p = 0.0001) and the neuromuscular scoliosis group (p = 0.007). Patients with congenital scoliosis had a much higher rate of reoperation when compared with the idiopathic scoliosis group (P = 0.0001) (Table 3 and Figure 4).

**Table 3:** Diagnosis and Reoperation Rates.

Diagnosis	Reoperation	No Reoperation	Rate	Total
Congenital scoliosis	34	1005	3.27%	1039
Idiopathic scoliosis	14	1241	1.12%	1255
Neuromuscular scoliosis	2	180	1.10%	182
Neurofibromatosis with scoliosis	2	77	2.53%	79
Degenerative scoliosis	2	99	1.98%	101
Marfan syndrome with scoliosis	4	47	7.84%	51
Others	1	50	1.96%	51
Total	59	2699	2.14%	2758

Others include syndrome-related scoliosis, ankylosing spondylitis, achondroplasia with scoliosis.

We found a significant difference in the rates of reoperation based on gender. The reoperation rate was 3.0% (26/833) for males and 1.71% (33/1925) for females (P = 0.019). Patients were divided into two age groups: ≤19 years and >19 years. We found significantly fewer reoperations in the ≤19-year-old group compared with the >19-year-old group (p = 0.006) (Table 4).

**Table 4:** Risk factors for reoperation.

	Reoperation within 30 days	No reoperation	Total	Reoperation rate (%)	P
Sex					0.019
Male	26	807	833	3.12	
Female	33	1892	1925	1.71	
Age					0.006
≤19years	42	2279	2321	1.81	
>19 years	17	420	437	3.89	
Procedure					0.006
Osteotomy	21	562	583	3.74	
No Osteotomy	38	2137	2175	1.78	
Kyphosis					0.613
Kyphosis	2	64	66	3.13	
Without kyphosis	57	2635	2692	2.16	
surgical approach					0.634
Anterior	4	152	156	2.56	
Posterior	49	2351	2400	2.04	
Combined	6	196	202	2.90	

The reoperation rate by surgical approach is listed in Table 4. There was a non-statistically significant increase in reoperation rates for patients having combined anteroposterior segmental fusion when compared with anterior or posterior fusion alone (p = 0.634). When patients were group according to whether osteotomy was performed in the operation, osteoectomy group had a higher rate of reoperation compared to non- osteoectomy group (p = 0.01). The most common reasons for repeat surgery in patients who underwent posterior instrumentation and fusion were implant failure, pulmonary complications, and wound infection. For anterior surgery, the most common reason for repeat surgery was pulmonary complications. For combined anteroposterior surgery, the most common reason for reoperation was implant failure.

Analysis of the reasons for neural complications indicated that patients with Marfan syndrome with scoliosis had the highest rate of neural complications and had a significantly higher rate when compared to the idiopathic scoliosis group (p = 0.01). The congenital scoliosis group also had a significantly higher rate when compared to the idiopathic scoliosis group (p = 0.03) (Table 5). When patients were group according to whether osteoectomy was performed during the operation, the osteoectomy group had a higher rate of reoperation for neural complications compared to non-osteoectomy group (p = 0.01) (Table 5).

**Table 5:** Risk factors for reoperation for neurologic deficit.

	Reoperation for neurologic deficit within 30 days	Reoperation within 30 days	No reoperation	Total	Reoperation rate (%)	P
Procedure						0.01
Osteotomy	6	21	562	583	1.03	
No Osteotomy	3	38	2137	2175	0.14	
Diagnosis						0.09
Congenital scoliosis	6	34	1005	1039	0.58	0.03★
Idiopathic scoliosis	1	14	1241	1255	0.14	
Neuromuscular scoliosis	0	2	180	182	0	
Neurofibromatosis with scoliosis	0	2	77	79	0	
Degenerative scoliosis	1	2	99	101	0.99	
Marfan syndrome with scoliosis	1	4	47	51	1.96	0.01★
Others	0	1	50	51	0	

★ When compared to idiopathic scoliosis group.

## Discussion

Unplanned reoperations represent major events for patients and have considerable impacts on the healthcare system, especially when the reoperations occur within 30 days after the initial surgery [14]–[16]. Reoperation rate has been used as a criterion for evaluating surgical department practice and even overall hospital care [17]. Unplanned reoperations increase the burden on the healthcare system as they result in operating theatre occupation, affect surgical waiting lists and lead to longer hospital stays and therefore higher costs [18]. These procedures can have an impact on staff trust and self-confidence. Although several studies have analyzed the rates of long-term reoperation after primary surgery for spinal deformity, the incidence and factors contributing to unplanned reoperation within 30 days of fusion surgery for spinal deformity have not been previously reported. This retrospective study is the first to provide data on the incidence and factors contributing to unplanned reoperation within 30 days of fusion surgery for spinal deformity. The overall unplanned reoperation rate within 30 days of primary surgery for spinal deformity in our study was 2.1%. There was no difference in patients' characteristics regarding age, gender, maximum Cobb measurement, and mean number of levels fused. The most common reasons for repeat surgery within 30 days of initial surgery were implant-related failures, pulmonary complications, wound infection, and neurologic deficits. Age older than 18 years, congenital scoliosis, syndrome-related spine deformity and inclusion of osteoectomy in the operation were risk factors for unplanned reoperation within 30 days of fusion surgery for spinal deformity.

A number of studies have been published investigating the rate of surgical revisions after spinal deformity surgery. In a retrospective review of patients who underwent instrumented spinal fusion for primary adult spinal deformity, Pichelmann et al reported a revision rate of 9.0% with a mean time to revision of 4.0 years with 45% of the revisions occurring within the first 2 years [13]. The most common reason for revision was pseudarthrosis, with wound infection as the second most common reason. In our study, implant-related failure was the most common reason for revision. Implant failure remains a major surgical challenge in the correction of spinal deformity. Aside from technical error and improper instrumentation, poor quality of bone and its structure are the main causes of implant failure. In our series, the implant-related failure complications requiring reoperation included hook pull-out, improper implant location, pedicle screw loosening, and screw cap loosening.

Postoperative spine infection is a complication that may have a significant impact on clinical outcome and is an important consideration in surgical decision-making [19], [20]. Therefore, optimal prevention and management of infection reflects not only a well-coordinated multidisciplinary team and an experienced surgeon but also the quality of the entire institution. Many series report postoperative infections as the most prevalent indication for repeat surgery, with the prevalence of infection after scoliosis surgery being 4.7% [21], [22]. Our series contained five superficial wound infections and 13 deep wound infections that required repeat surgery. In line with previous studies, all of the infections were observed in patients whose index procedure was posterior spinal instrumentation and fusion. No infections occurred following anterior spinal instrumentation.

Previous studies have reported that no reoperations were needed for neurologic complications [23]. In our series, however, 9 revisions were due to neurologic deficit: 6 of which were due to paraplegia and 3 were due to nerve root injury. Of the 6 patients who presented with paraplegia, 4 presented with complete paralysis of the lower extremity and 2 presented with incomplete paralysis of the lower extremity.

Pulmonary complication was the next most common reason for readmission [23], [24]. This complication includes hydrothorax, hemothorax, pneumothorax and chylothorax. Surgical technique has a significant influence on postoperative pulmonary complications. Anderson et al. found that the incidence of postoperative pulmonary complications in patients who underwent anterior fusion was 3 times that in patients who underwent posterior fusion. In our study, however, we found no significant difference between the surgical approaches.

It is important to be able to identify the risk factors for developing complications that could require revision surgery within 30 days after initial surgery; the precise knowledge of reoperation risks is valuable information for both patients and surgeons. Previous studies have shown that older age, increased body mass index, and osteopenia are significant risk factors for developing a complication [14], [25]. Mok et al [9] identified risk factors for infection (age, diabetes, hypothyroidism, and surgeon's experience) and adjacent segment decompensation (age, smoking, and cardiac comorbidity). The current study represents, to our knowledge, the first analysis of risk factors for readmission within 30 days after initial surgery for spinal deformity. In accordance with the data of previous authors [9], patient age over 18 years conferred a risk of developing a complication that required reoperation 1.8 times higher than those younger than 18. Congenital scoliosis and Marfan syndrome with scoliosis presented a significantly higher revision rate than other spinal deformities. Furthermore, with regard to the reasons for neural complications, patients with congenital scoliosis and syndrome-related scoliosis also had a higher rate when compared to the other groups. Moreover, no statistically significant difference was found in the reoperation rate based on the surgical approach. Patients whose surgery included osteoectomy had a higher rate of reoperation compared to the non-osteoectomy group.

In conclusion, this is the first study to provide insight into the incidence and factors contributing to unplanned reoperation within 30 days of fusion surgery for spinal deformity. The rate of unanticipated revision surgery within 30 days after primary surgery is 2.1%. The reasons for reoperation included implant-related failures, pulmonary complications, infections, neurologic deficit and coronal plane imbalance. The importance of comorbidities often present in this patient population is highlighted by the significantly elevated risks found with increasing age, congenital scoliosis and Marfan syndrome with scoliosis as well as inclusion of osteoectomy in the operation. The information contained in this report will assist surgeons with preoperative risk stratification and facilitate discussions with patients to make informed choices in surgical decision-making.

## Materials and Methods

### Ethics statement

All of these protocols were approved by the Clinical Research Ethics Committee of the Peking Union Medical College Hospital. Data were obtained from surgical patients after obtaining approval from the Clinical Research Ethics Committee of the Peking Union Medical College Hospital and fully informed written consent from the patients or patients' parents.

### Patient Population

We retrospectively reviewed a prospectively collected database at our institution to identify patients who underwent a definitive spinal fusion between 1998 and 2012 for a diagnosis of spinal deformity. Spinal deformity was defined as any major coronal, sagittal, or combined deformity requiring instrumented fusion. We excluded patients who underwent spinal deformity surgery for other etiologies, such as acute vertebral fracture, spinal tumor, active infection, paraplegia, and those who had previously undergone primary surgery. The length of follow up for each patient was more than 30 days. A revision or reoperation surgery within 30 days of fusion surgery was defined as any unanticipated return to the operating room after the index procedure. All surgeries were performed by the senior author.

### Data Collection

Patients' names, dates of birth, genders, medical record numbers, diagnoses, dates of surgery, ages at surgery, approaches (anterior, posterior, or combined), and types of implants were recorded in the surgical logs. All subsequent reoperations at our institution were also recorded, and chart reviews were performed to ensure that no patients had undergone a known reoperation at another institution. The primary reason for the reoperation was recorded. If, for some reason, there appeared to be two or more possible factors responsible for further surgery, the predominant factor was chosen as the reason for reoperation and used in subsequent analyses. The reasons for reoperation were categorized into one of the following groups: (1) implant failure, (2) wound infection, (3) neurologic deficit (4) pulmonary complications, or (5) coronal plane imbalance. The decision to reoperate was based on the clinical judgment of the treating surgeon.

### Statistical Analysis

Statistical analyses were performed using Student's *t*-test for continuous variables and the Fisher exact test and *X* ^2^ test for categorical variables. A P-value of <0.05 defined significance.
